# Lipophilic arsenic compounds in the cultured green alga *Chlamydomonas reinhardtii*

**DOI:** 10.1007/s00216-023-05122-7

**Published:** 2024-01-08

**Authors:** Andrea Raab, Jinyu Zhang, Ying Ge, Fernando Fernández-Mendoza, Jörg Feldmann

**Affiliations:** 1 TESLA – Analytical Chemistry, University of Graz, Universitätsplatz 1, 8010 Graz, Austria; 2 College of Resources and Environmental Sciences, Nanjing Agricultural University, 1 Weigang, Nanjing, China; 3 School of Biology, University of Graz, 8010 Graz, Austria

**Keywords:** *Chlamydomonas reinhardtii*, Arsenic, Arsenolipids, As phytol, Arsenosugars, Mass spectrometry, HPLC, Algae

## Abstract

In this study, arsenic (As) speciation was investigated in the freshwater alga *Chlamydomonas reinhardtii* treated with 20 μg/L arsenate using fractionation as well as ICP-MS/ESI-MS analyses and was compared with the known As metabolite profile of wild-grown *Saccharina latissima*. While the total As accumulation in *C. reinhardtii* was about 85% lower than in *S. latissima*, the relative percentage of arsenolipids was significantly higher in *C. reinhardtii* (57.0% vs. 5.01%). As-containing hydrocarbons and phospholipids dominated the hydrophobic As profile in *S. latissima*, but no As-containing hydrocarbons were detectable in *C. reinhardtii*. Instead for the first time, an arsenoriboside-containing phytol (AsSugPhytol) was found to dominate the hydrophobic arsenicals of *C. reinhardtii*. Interestingly, this compound and its relatives had so far been only found in green marine microalgae, open sea plankton (mixed assemblage), and sediments but not in brown or red macroalgae. This compound family might therefore relate to differences in the arsenic metabolism between the algae phyla.

**Supplementary Information:**

The online version contains supplementary material available at 10.1007/s00216-023-05122-7.

## Introduction

Arsenic is a highly toxic metalloid found ubiquitously in the environment in the form of inorganic and organic salts, as well as in gaseous form. Its release into the environment has been increased by human activity, especially by the extraction of mineral resources, and it is a cause of growing ecotoxicological concern, especially in aquatic ecosystems. The determination of arsenic-containing compounds in biological materials has received significant attention across different areas of research. Toxicological surveys mostly focused in evaluating the toxicity of natural and man-made inorganic arsenic species [1], while biochemistry and analytical chemistry centered on non-targeted determination of arsenic species found as organoarsenicals in nature. Organoarsenicals comprise a variety of hydrophilic compounds including monomethylarsonic acid (MMA), dimethylarsinic acid (DMA), and arsenoribosides (AsSugars) as well as a large range of lipophilic compounds (AsLipids). AsLipids can be further categorized into arsenic-containing hydrocarbons (AsHC) [2, 3], fatty acids (AsFA) [4–7], long-chain alcohols (AsOH) [2], phosphatidylcholines (AsPC) [8] and ethanolamine (AsPE) [8], arsenoriboside-containing phospholipids (AsSugPL) [9–11], ether-phospholipids (AsEP) [12], and phytyl-lipids (AsSugPytol) [13, 14]. Understanding the distribution of organic arsenic species and their role in detoxification is key to understanding the cycling of arsenic within ecosystems. Among eukaryotes, arsenic species found in marine algae have received particular attention due their generally relatively high concentrations and high variability [15]. The high diversity of arsenic species found in algae results from the extreme taxonomic and phylogenetic diversity found within them [16, 17], but at the same time it is strongly influenced by the pervasiveness of non-genealogical bonds [18] between distantly related genomes, also identified in arsenic detoxification pathways [19–21] as a result of endosymbiosis or horizontal gene transfer [22]. AsSugars are in general the major hydrophilic arsenic species present in marine algae, but a wide variety of AsLipids has also been identified to be of relevance in different algal groups. AsHCs have been found to be the major arsenolipid species in marine macroalgae, fish, and fish derivatives [10, 23]. AsFAs on the other hand have predominantly been found in fish [4, 23] and molluscs [12]. Both AsFA and AsHC are closely related to naturally occurring fatty acids that are not associated with As. The presence of AsSugPL seems to be limited to algae, and organisms that feed directly on them like mussels [12]. While algae also produce arsenic-containing sugars [24], AsSugPL has been identified as an important lipophilic arsenical in both marine macro- and microalgae, where they may be produced in response to oxidative stress [25]. AsSugPhytols have, so far, been identified in the marine green microalgae *Dunaliella tertiolecta* [13] and *Picocystis* strain ML [26] as well as in environmental samples of marine plankton [27]. In contrast, arsenic-containing lipids in freshwater or terrestrial microalgae have received less attention and only one publication studying arsenolipids in a freshwater cyanobacterium exists [28] but none for green freshwater algae.

Here we show the distribution of lipophilic and hydrophilic arsenic species in the freshwater/terrestrial alga *Chlamydomonas reinhardtii* exposed to arsenate (As(V)). The results were also compared to the known As metabolites of *Saccharina latissima*, a brown marine macroalgae often studied in the literature [10, 35].

## Methods

### Chemicals

Throughout the experiments, 18 MΩ cm water (Millipore, Merck) was used. Methanol (MeOH), dichloromethane (DCM), hexane (Hex), and acetone were of HPLC-grade from Roth (Austria). Formic acid p.a. was from Sigma (Germany). Arsenic standard solution (1000 mg/L) was from Roth (Austria) and DMA standards were prepared by dissolving appropriate amounts of Na_2_-DMA (Sigma, Germany) in water. For total arsenic determination IRMM-CD200 (certified As concentration 55 ± 4 mg/kg), an algae-based material was used as quality control. Additionally, freeze-dried and finely ground *Saccharina latissima* (SL) collected in the wild at Stonehaven, Scotland, in 2019 was used for comparison and to support identification of As species, since the As species in it were identified before [10]. Chemicals used for the algal culture, namely tris-acetate-phosphate (TAP) medium (Table S1), were of analytical grade or better and purchased from Sinopharm Chemical Reagent Co. (China).

### Algae culture

*Chlamydomonas reinhardtii* (strain CC-125) was bought from the Chlamydomonas Resource Center located at the University of Minnesota [29]. Algae were grown, at two different occasions in duplicate, mixotrophically in tris-acetate-phosphate (TAP) medium (for details, see Table S1) at 25 ± 2 °C, pH of 7.0 ± 0.1, light intensity of 3000–3500 lx (cool white light), and alternate light and dark periods of 12 h and 12 h, respectively. In the middle of the exponential growth phase, As(V) was added to the growth medium (300 mL) and the 20 μg/L As(V) treatment lasted for 7 days. The algal sample without As(V) addition was set up as control for growth comparison only. The optical density at 680 nm (OD680) value of *C. reinhardtii* was measured using a microplate reader. It was around 0.10 initially and reached between 1.44 and 1.47 at the end of culture when no As(V) was added. The growth was reduced by about 15% in the As(V) treatment since the OD values increased only to about 1.2 (Fig. S1). The microalgal pellets were collected from the solution through centrifugation (6000 g/min; 2 min). The pellets were rinsed three times with PBS solution and deionized water to remove adsorbed As on the cell surface. The samples were lyophilized and stored for subsequent As analyses (total weight for each sample was approximately 500 mg). From the two biological replicates at each occasion, three technical replicates each were created for analysis (*n* = 12).

### Total arsenic determination

Samples (10 mg) were weight to ± 0.02 mg in digestion vials and digested using 2 mL conc. HNO_3_ and 2 mL 18 MΩ cm water using an UltraClave IV (Analytix, Austria). All samples, including the reference material CD-200 and *S. latissima* (SL), were prepared in triplicate. The UltraClave IV was pre-pressurized to 40 bar using argon. The temperature was increased to 250 °C and held there for 30 min before cooling down. Samples for total arsenic in extracts and residual As in the pellet were weighed to ± 0.02 mg into digestion vials and digested using 1 mL HNO_3_ and 3 mL H_2_O following the same microwave protocol.

The clear digested samples were transferred to 15 mL PP vials (Fisher, Germany) and diluted with water to 10 (± 0.0002) g. Arsenic was determined in the diluted digests using an 8900 Agilent ICP-MS/MS with 5 µg/L Ge as internal standard (constantly added via a T-piece). Quantification was done via external calibration using appropriately diluted standards in 10% HNO_3_. All concentrations are presented as per dry mass (d.m.) algae.

### Extraction of algae for quantitative arsenic speciation

Samples (20 mg) were weighed to ± 0.02 mg into 2-mL reaction vials (PP) and extracted on a shaker/incubator cooled to 10 °C overnight with 1 mL hexane followed by a second extraction with 1 mL hexane for 2 h. The two hexane fractions were combined in digestion vessels and dried before digestion for total As determination; they were not used for speciation analysis. The hexane-extracted pellet was re-extracted on a shaker/incubator cooled to 10 °C using 1 mL DCM/MeOH (2:1) overnight and a second extraction with 1 mL DCM/MeOH (2:1) for 2 h. The two DCM/MeOH extracts were combined, part of this extract (50 mg) was added to digestion vials and dried before digestion for total As determination, and the rest was dried in a speed-vac (Christ RVC 2–33 CO plus, Germany) for speciation analysis. The residual pellet was extracted two times with 1 mL water (each for 2 h at 20 °C). The two water extracts were combined and freeze dried after removal of 50 mg extract solution for total As determination. All extracts were stored dry at − 20 °C.

### Quantitative speciation of water-soluble arsenic species

The freeze-dried water extracts were diluted with water to 0.5 g (± 0.02 mg). The water-soluble species were separated with 20 mM ammonium phosphate pH 6.2 on an anion exchange column (Hamilton PRP-X-100 4.6 × 250 mm) with a flow rate of 1 mL/min at 40 °C column temperature using an Agilent 1100 HPLC, injection volume 20 µL. An Agilent 7700 ICP-MS, optimized for sensitivity, was used in He mode (4 mL He/min) as detector for ^75^As. Additionally m/z 53 and m/z 77 were monitored; no interference from chloride was noticed. DMA solutions diluted appropriately with water were used as standards for external calibration. Identification of species was done via retention time comparison with standards.

### Quantitative speciation of DCM/MeOH soluble As species

The DCM/MeOH soluble fraction was dissolved in 0.5 g (± 0.02 mg) methanol. For separation, an ACE C18 Excel 3 column (4.6 × 250 mm) was used. A linear gradient from 0 to 100% MeOH (0.1% formic acid (v/v)) in 20 min with hold for 25 min was used. Solvent A was 0.1% formic acid (v/v) in water. The flow rate was 1 mL/min, injection volume 20 μL. The HPLC system used for coupling with ICP-MS/MS (8800 Agilent, Agilent, Germany) and ESI-MS (6460 triple quad MS) was an Agilent 1260, flow split 1-part ICPMS/MS and 9-parts ESI-MS (1:10, AS model 620, ASI, USA). The ICP-MS/MS was used with a narrow-bore torch (1 mm), platinum cones, and 30% oxygen (~ 0.3 mL/min) as reaction gas. Isotopes monitored were phosphorus (^31^P- > ^31^P^16^O), sulphur (^32^S- > ^32^S^16^O), ^74^Ge (continuous internal standard in 5% acetone added via an additional HPLC pump at 0.8 mL/min), and arsenic (^75^As—> ^75^As^16^O). The ICP-MS/MS data were used for quantification of As-containing species, whereas the ESI-MS was used in positive scan mode (spray voltage 4.5 kV, capillary temperature 335 °C, sheath gas 400 °C) scanning from 150 to 1300 m/z for identification in combination with data from high-resolution ESI-MS measurements of the same samples. The instruments were optimized for sensitivity before measurement. The results from ESI-MS (low-resolution) were compared to high-resolution measurements done with a MaXis II (qTOF from Bruker, Germany) with the same separation conditions (flow split 1:5 ICP-MS/MS set-up as described above) for detailed identification of species. The qTOF was used in positive mode with 4.5 kV spray voltage, capillary temperature 350 °C, mass range 100–1500 m/z, and optimized as required for sensitivity. Ammonium formate clusters were used for mass calibration before each injection and C_12_H_19_F_12_N_3_O_6_P_3_ (m/z 622.0290) used as continuous lock-mass. All concentrations are presented as per dry weight algae.

## Results

### Total arsenic content in algae and specific fractions

**Fig. 1:**
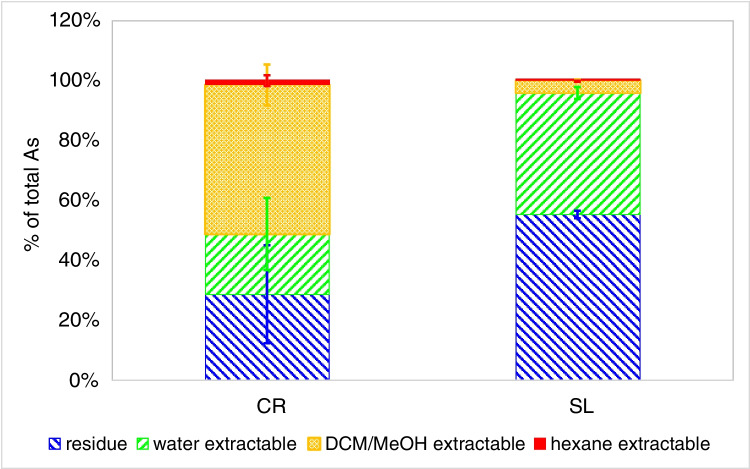
Hexane, DCM/MeOH, and water-extractable arsenic and residual arsenic in % of total arsenic in *C. reinhardtii* (CR, *n* = 12) and *S. latissima* (SL, *n* = 3)

The recovery of the reference material CD-200 with measured 51 ± 3.2 mg As/kg was within the certified range of 55 ± 4 mg As/kg (Table S2)*. Saccharina latissima* contained 81 ± 8.7 mg As/kg d.m. (Fig. 1, Table S2), which is very similar to concentrations found previously [10]. The detection limit for total arsenic was 0.01 µg As/kg sample determined using replicate blank measurements as 3 times the standard deviation (*n* = 10). *C. reinhardtii* (*n* = 4) cultivated under identical conditions at two different times and exposed to 20 µg As/L for 7 days contained about 11 mg As/kg algae d.m. (Fig. 1, Table S2). Comparison of the 2 different time points showed no statistical significant difference between the cultures (*T*-test *p* = 0.22). In a previous study with the same As(V) treatment, the As accumulation in *C. reinhardtii* was 13.8 mg As/kg algae d.m. [30]. Another unicellular green algae *Dunaliella tertiolecta*, a seawater alga, was found to contain about 18 mg As/kg algae d.m. when exposed to 15 µg As/L [13]. Duncan et al. reported for the same algae at exposure concentrations of 2 µg As/L with variable exposure time total As concentrations between 6 and 10 mg As/ kg d.m. [31, 32]. *Chlorella vulgaris* exposed to 10 µg As/L was found to contain about 18 mg As/ kg algae d.m. [33]. These concentrations are similar to the ones found in our study.

The total extraction efficiency (sum of all extraction media used) for *S. latissima* was about 52%, indicating that nearly half of the As present was not extractable by either solvent used, as confirmed by As determination in the residue. Most of the extractable As in *S. latissima* was hydrophilic arsenic (47.6 ± 5.5) %, with (5.0 ± 0.22) % soluble in DCM/MeOH and less than 0.1% in the hexane fraction (Fig. 1, Tables S2 and S3). These values are similar to literature values [10].

About 20% of the As present in *C. reinhardtii* was not extractable by either hexane, DCM/MeOH, or water (Fig. 1, Tables S2 and S3)*.* The distribution of extractable As in *C. reinhardtii* was different compared to *S. latissima* with the majority of As found in the DCM/MeOH fraction (55.3 ± 5.7) % for *C. reinhardtii* (Fig. 1, Tables S2 and S3). The hexane soluble fractions of *C. reinhardtii* contained 1.64 ± 1.9% of the total arsenic; one of the replicate cultures contained significantly (*T*-test *p* < 0.005) more hexane-extractable As than the other three (5% vs. 0.6% of total As). The reason for this is at the moment unknown. Hydrophilic As compounds contributed about 22% to the total As in *C. reinhardtii* whereas in *S. latissima* nearly 50% of the total As was water-soluble (Fig. 1, Tables S2 and S3). Duncan et al. [31] found that *D. tertiolecta* exposed to As(V) contained about 38% of the total As as lipophilic As, 7% as hydrophilic As, and about 55% residual As, whereas Forster et al. [34] determined a lipophilic contribution of 29–38% and a hydrophilic contribution of 20–29% in *D. tertiolecta.* For *Phaeodactylum tricornutum*, they found 4.2–9% lipophilic As and 26–36% hydrophilic As [34]. *C. reinhardtii* seems to transform relative more As(V) to lipophilic As than *D. tertiolecta* and generally to contain more extractable As, but this is very likely species dependent and also influenced by the exact culture conditions used.

### Speciation of hydrophilic arsenic compounds

**Fig. 2:**
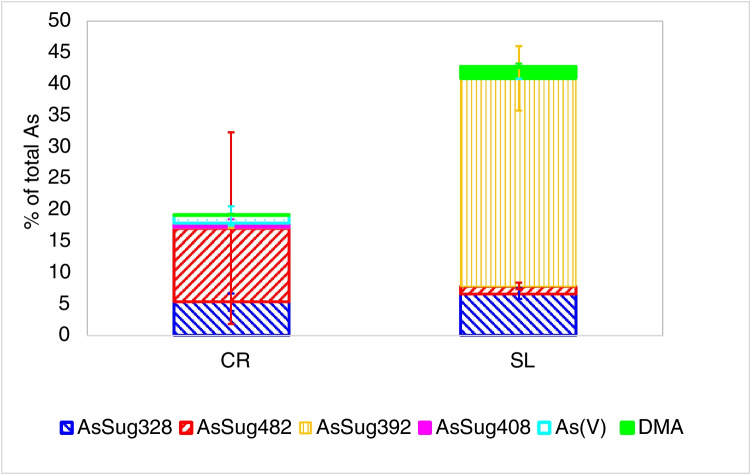
Amount of different water soluble As species relative to total arsenic in water extract of *C. reinhardtii* (CR, *n* = 12) and *S. latissima* (SL, *n* = 3), chromatographic conditions: PRP X 100, 20 mM ammonium phosphate buffer pH 6.2, detection/quantification: ICP-MS

*S. latissima* contained predominantly AsSug392 and AsSug328 besides DMA and AsSug482, each contributing between 1 and 33% to the total arsenic present in *S. latissima* (Fig. 2, Table S4). The species distribution is similar to the one reported by Petursdottir et al. for fresh *S. latissima* [35].

The water-soluble As species in *C. reinhardtii* contributed between 6 and 45% to the total arsenic depending on culture (Fig. 1, Table S3). AsSug328 was one of the major hydrophilic compounds in *C. reinhardtii* (~ 5% total As). Miyashita et al. [36] found during incubation experiments with 11.2 mg As(V)/L for 24 h that *C. reinhardtii* synthesized predominantly AsSug328. Under the growth conditions used in our experiments, the relative amount of AsSug482 was similar to AsSug328, but the AsSug482 concentrations were highly variable (Table S4). Although in the 24-h exposure experiments of Miyashita et al. significant less AsSug482 than AsSug328 was produced, these authors also describe a high variability of AsSug482 amounts present under their culture conditions [36]. This may indicate that AsSug482 content is dependent on the activity of enzymatic pathways transforming this compound into AsPLs.

DMA, inorganic As, and the sulphur-containing sugars AsSug408 and AsSug392 were minor compounds in *C. reinhardtii*, with concentrations around the detection limit (Fig. 2, Table S4). The chromatographic quantification limit here was about 0.01 mg As/kg sample estimated from blank injections and the lowest standard concentrations used. A typical chromatogram for the separation of the hydrophilic compounds detected in *C. reinhardtii* is shown in Fig. S2.

### Speciation of hydrophobic arsenic species

**Fig. 3:**
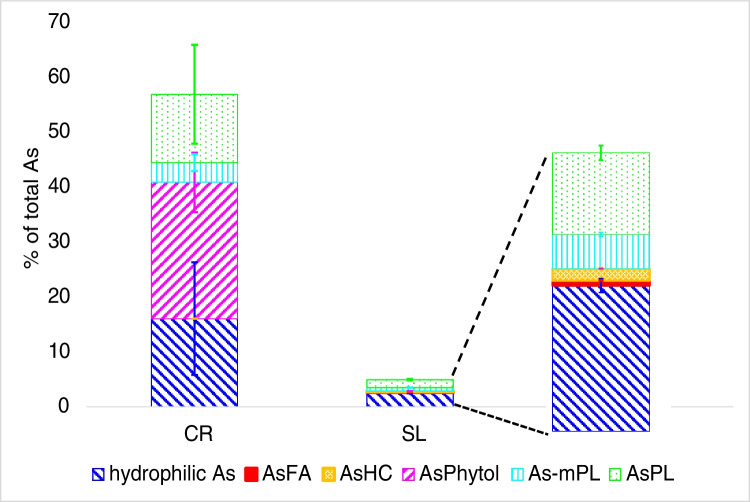
Amount of different hydrophobic As species relative to total arsenic in *C. reinhardtii* (CR, *n* = 12) and *S. latissima* (SL, *n* = 3), chromatographic conditions: RP-C18 separation, water/methanol gradient; detection: ICP-MS/MS and ESI-MS (flow split 1:10)

**Fig. 4:**
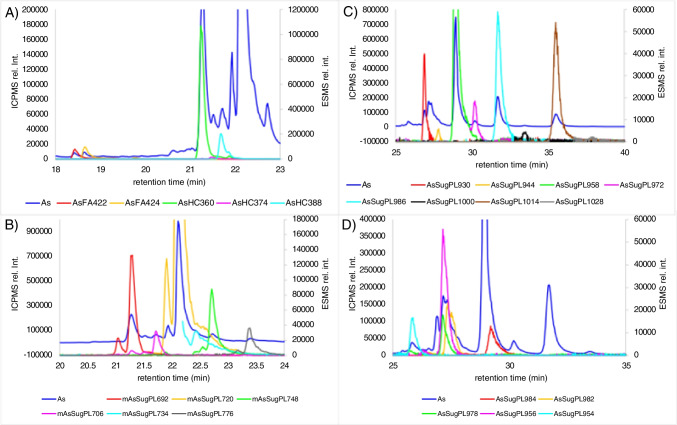
*S. latissima* ICPMS arsenic trace (blue) overlayed by extracted ion chromatograms (EIC ± 5 ppm) of ESMS. *A* AsFA and AsHC. *B* Mono-AsSugPL. *C* Saturated AsSugPL. *D* Unsaturated AsSugPL. Chromatographic conditions: RP-C18 separation, water/methanol gradient detection: ICP-MS/MS (As as AsO^+^) and ESI-qTOF (for all (M+H)^+^ detections) with a flow split 1:5; the signals for MeOH-adducts were removed from the EICs; for mass accuracy and fragmentation, see Table S6 and Fig. S3-S12

*S. latissima* extracted with DCM/MeOH contained among others small amounts (< 0.1% total As) of the two unsaturated fatty acids AsFA422 and AsFA424 identified in this species by Raab et al. [10] as well as AsHCs and AsSugPLs found in the same study and by Petursdottir et al. [34] (Figs. 3, 4A, Table S5).

AsHCs constituted 0.21% of the total arsenic content in *S. latissima*. About 60% to the total AsHC content was contributed by AsHC360, followed by AsHC388, and AsHC374 was a minor compound among the AsHCs (Fig. 4A). AsSugPL and mono-AsSugPL constituted about 2% of the total arsenic in *S. latissima*. Among the AsSugPLs, AsSugPL958 (C16:0/C16:0) was the dominant compound (~ 33%) followed by the saturated AsSugPL986 (C18:0/C16:0), AsSugPL 1014 (C20:0/C16:0), AsSugPL 930 (C14:0/C16:0), and AsSugPL 972 (C17:0/C16:0) (Fig. 4C). Among the unsaturated, AsSugPL982 (C18:2/C16:0) was the dominant one, closely followed by AsSugPL 978 (C18:4/C16:0 or C18:2/C18:2) and AsSugPL 984 (C18:1/C16:0) (for species identification and distribution, see Fig. 4D, Table S5, Table S6, and Fig. S3–S12). This batch of *S. latissima* contained more mono-acylated AsSugPL with mAsSugPL720 (C16:0) being the dominant form, followed by mAsSugPL692 (C14:0), and minor compounds were mAsSugPL734 (C17:0), mAsSugPL748 (C18:0), mAsSugPL776 (C20:0), and mAsSug706 (C15:0) (Fig. 4B) than the samples used in previous publications [2, 4]. Spectra of all compounds which were confirmed not only by accurate mass but by fragmentation can be found in the supplement (Figures S3 to S12).

**Fig. 5:**
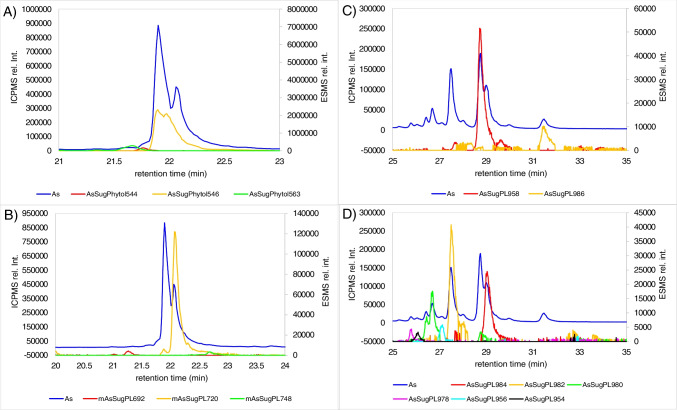
*C. reinhardtii* ICPMS arsenic trace (blue) overlayed by extracted ion chromatograms (EIC ± 5 Δppm) of ESMS. *A* AsSugPhytol. *B* Mono-AsSugPL. *C* Saturated AsSugPL. *D* Unsaturated AsSugPL. Chromatographic conditions: RP-C18 separation, water/methanol gradient detection: ICP-MS/MS (As as AsO^+^) and ESI-qTOF (for all (M+H)^+^ detections) with a flow split 1:5; the signals for MeOH-adducts were removed from the EICs; for mass accuracy and fragmentation, see Table S6 and Fig. S13-S18

The DCM/MeOH extract of the *C. reinhardtii* contained about 6 mg As/kg d.m. algae. In contrast to *S. latissima*, no traces of AsFA and AsHCs were found in *C. reinhardtii*. AsSugPL and mAsSugPL were both present in *C. reinhardtii* (Table S5, Fig. 5C, D). Compared to *S. latissima*, the number of AsSugPL was reduced in *C. reinhardtii*, only AsLipids with fatty acids C16:0, C16:1, C18:1, C18:2, C18:3, and C18:4 were found (for identification and fragmentation, see Table S6 and Fig. S13-S18). Fatty acids containing 16 or 18 carbon atoms seem to be naturally the major fatty acids found in *C. reinhardtii* [37]. This could indicate that the biosynthetic pathway for fatty acids is used to produce the AsSugPL.

The majority of hydrophobic arsenic in *C. reinhardtii* belonged to the compound-group AsSugPhytol, which was not found in *S. latissima* (Fig. 3, Fig. 5A, Table S5). This compound-group was first shown to occur in the marine alga *D. tertiolecta* [14] and has since been detected in sediments and open-ocean plankton samples [27, 38]. In *C. reinhardtii*, the dominant species among them was AsSugPhytol546. Beside this, small amounts of AsSugPhytol544 and AsSugPhytol563 were detected (Fig. 5A). The phytyl-sidechain of AsSugPhytol546 is identical to the phytyl-sidechain of chlorophyll, which may explain its prominence in *C. reinhardtii* as a green alga. Spectra of all compounds which were confirmed not only by accurate mass but by fragmentation can be found in the supplement (Figures S13 to S18).

## Discussion

The cultures of *Chlamydomonas reinhardtii* exposed to arsenate for 7 days metabolized about 80% of the absorbed arsenic into a variety of extractable methylated arsenic compounds. The hydrophilic compounds found are coherent with previous findings in *C. reinhardtii* CC125 [39]. The arsenoribosides AsSug328 and AsSug482 were dominant in both strains (> 60% of hydrophilic As, Fig. 2), both being potentially precursors and/or breakdown products of AsSugPL. These findings contrast with those of *Chlorella vulgaris* which did not metabolize significant amounts of arsenic to methylated arsenic compounds when exposed to high As concentrations (10–1000 mg As(V)/L) [40], although small amounts of AsSug328 and AsSug482 were detected besides DMA. The similarity of our results obtained exposing the cultures to 20 µg As(V)/L with those previously published for *C. reinhardtii* CC1 [39] using 200 µg As(V)/L contrasts with the observations made in the marine species *D. tertiolecta*, in which between 1 and 54% of the hydrophilic arsenic was present as AsSug328 and AsSug482 depending on the culture conditions [15]. The ability to synthesize AsSugars at low arsenic concentrations seems to be widespread in marine and freshwater microalgae, at least within the Chlorophyta.

More than half of the arsenic in *C. reinhardtii* was extractable by DCM/MeOH in contrast to the mere 5% extracted from the brown alga *S. latissima*. The low proportion of As extractable with DCM/MeOH in *S. latissima* is similar to that published by Petursdottir et al. [35], but half of that determined by Raab et al. in the same species [10], reflecting either seasonal variability or an effect of sample storage. The results found for *C. reinhardtii* fit with the observations made in green marine macroalgae for which Thomson et al. found between 19 and 44% of the total arsenic in to be present in lipophilic compounds [41]. The same authors reported lower amounts of AsLipids in red algae which are phylogenetically related to the chloroplast of brown algae.

**Fig. 6:**
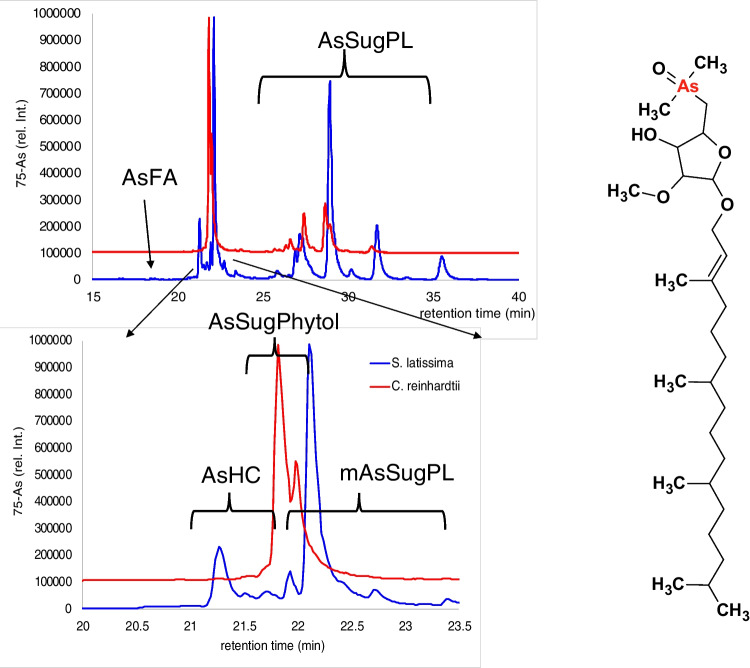
Comparison of the lipophilic arsenic species in *C. reinhardtii* and *S. latissima*; 91AsO-signal from ICP-MS/MS, chromatographic conditions: RP-C18 separation, water/methanol gradient detection: ICP-MS/MS and ESI-qTOF (flow split 1:5); right side: structure of AsSugPhythol546

The major lipophilic arsenic compounds in *C. reinhardtii* were AsSugPhytols (Fig. 6) and it did not contain any detectable AsHCs or AsFAs, common in *S. latissima* [10]. The lack of AsHCs in *C. reinhardtii* also contrasts with the findings of Glabonjat et al. for *D. tertiolecta* [13], where small amounts of AsHCs were found. AsSugPhytols were previously detected in *D. tertiolecta* as well as in environmental samples of saline environments dominated by microalgae, plankton, and sediment [13, 27, 37]. Contrastingly, AsSugPhytols have so far not been reported in marine macroalgae of the phyla Rhodophyta and Phaeophyta. When AsHC formation in macroalgae is accidental, then it should also occur in microalgae, which are well known to produce “normal” hydrocarbons with some strains exploited for biosynthetic production of fuel [42]. The fact that no AsHCs were synthesized by *C. reinhardtii* and that AsSugPhytol is to our knowledge not produced by members of the Rhodophyta and Phaeophyta phyla together with the study by Cruz-Morales et al. might be indications that the synthesis of As-containing lipids is at least partially genetically controlled [43]. The “normal” phytol is required for the synthesis of vitamins E and K [44] but does not seem to be involved in any sugar metabolism. Free phytol and free fatty acids form phytol-fatty acid esters, which seem to be intermediate storage forms for both metabolites [44]. Chlorophylls a and b (major chlorophylls in brown resp. green algae) both contain a phytyl-moiety. Phytol from chlorophyll degradation should therefore be present and available for the biosynthesis of AsSugPhytol in all algae families.

## Conclusion

The freshwater alga *C. reinhardtii* produced hydrophilic arsenoribosides (AsSug) and lipophilic arsenoriboside-phospholipids (AsSugPL) upon arsenate exposure. Both families of organoarsenicals are known to be widespread in marine macro- and microalgae, but AsSugPLs have been rarely studied so far in terrestrial or freshwater environments, so far only for a freshwater cyanobacterium (*Synechocystis* sp. PCC 6803) the arsenolipid content and distribution has been published [28]. AsSugPL982 and AsSugPL984 were found in this cyanobacterium, whereas no AsHCs were identified. Besides AsSugPL *C. reinhardtii* also produced significant amounts of AsSugPhytol. This class of compounds has so far only been detected in certain marine microalgae and marine plankton mixture, but not in freshwater organisms. Striking was also the lack of AsFA and AsHC formation in *C. reinhardtii*, whereas AsHC seemed to be omnipresent in marine micro- and macroalgae. This indicates that there are several yet unknown pathways involved in the biological cycling of arsenic in algae.

### Supplementary Information

Below is the link to the electronic supplementary material.

Supplementary file1 (PDF 402 KB)

## Data Availability

The datasets generated during and/or analyzed during the current study are available from the corresponding author on reasonable request.
