# Supplementary material for: Lipophilic arsenic compounds in the cultured green alga Chlamydomonas reinhardtii
Source: Anal Bioanal Chem. 2024 Jan 8;416(11):2809–18. doi: 10.1007/s00216-023-05122-7 (PMC11009773; doi:10.1007/s00216-023-05122-7)
Supplement: Supplementary file 1 — Supplementary file1 (PDF 402 KB) [file 216_2023_5122_MOESM1_ESM.pdf]

Electronic supplement

Lipophilic arsenic compounds in the cultured green alga *Chlamydomonas reinhardtii*.

Andrea Raab[1], Jinyu Zhang[2], Ying Ge[2], Fernando Fernández-Mendoza[3], Jörg Feldmann[1]

[1]TESLA – Analytical Chemistry, University of Graz, Universitätsplatz 1, 8010 Graz, Austria

[2] College of Resources and Environmental Sciences, Nanjing Agricultural University, 1 Weigang, Nanjing, China

[3]School of Biology, University of Graz, 8010 Graz, Austria

Table S1: TAP medium composition

Stock Solution	Components	Final Concentration (g/L)
Tris Base	$H_2NC(CH_2OH)_3$	2.42
TAP Salts	NH_4Cl	0.1875
	$MgSO_4·7H_2O$	0.05
	$CaCl_2·H_2O$	0.025
Phosphate Solution	$K_2HPO_4·3H_2O$	0.014148
	KH_2PO_4	0.0054
Trace Elements	$ZnSO_4·7H_2O$	0.022
	H_3BO_3	0.0114
	$MnCl_2·4H_2O$	0.00506
	$CoCl_2·6H_2O$	0.00161
	$CuSO_4·5H_2O$	0.00157
	$(NH_4)_6Mo_7O_{24}·4H_2O$	0.0011
	$FeSO_4·7H_2O$	0.00499
	$Na_2H_2EDTA·2H_2O$	0.05
Acetic Acid	CH_3COOH	1.05

Table S2: total arsenic determined after MW digestion of algae, sum of total extractable arsenic (hexane + DCM/MeOH + water) and residual arsenic, in *C. reinhardtii* (CR, n=12) cultivated at two different time points under identical conditions, *S. latissima* (SL) and reference material (CD-200); n=3

	total As (mg As/kg algae d.m.)	residual As (mg As/kg algae d.m.)	sum extractable As (mg As/kg algae d.m.)	% extractable As of total As
CR	11.0 ± 0.78	3.52 ± 2.0	8.69 ± 1.4	79.4 ± 13
SL	81.3 ± 8.7	52.7 ± 1.1	42.3 ± 1.9	52.6 ± 5.1
CD-200	51 ± 3.2			

Table S3: total arsenic determined after MW digestion of algae extracts, in *C. reinhardtii* (CR, n=12) cultivated at two different time points under identical conditions, *S. latissima* (SL) (n=3)

	Hexane extractable As (mg As/kg algae d.m.)	DCM/MeOH extractable As (mg As/kg algae d.m.)	water extractable As (mg As/kg algae d.m.)
CR	0.176 ± 0.203	6.08 ± 0.83	2.44 ± 1.5
SL	0.038 ± 0.006	4.02 ± 0.37	38.2 ± 1.9

Table S4: arsenic species determined in the hydrophilic fraction, in *C. reinhardtii* (CR, n=12) cultivated at two different time points under identical conditions, *S. latissima* (SL) (n=3); [% of total As]

	CR	SL
AsSug328 (mg As/kg algae d.m.)	0.58 ± 0.2<br>[5.2 ± 1.4]	5.32 ± 0.71<br>[6.6 ± 0.86]
AsSug482 (mg As/kg algae d.m.)	1.2 ± 1.6<br>[12 ± 15]	0.92 ± 0.5<br>[1.1 ± 0.52]
AsSug392 (mg As/kg algae d.m.)	0.022 ± 0.02<br>[0.20 ± 0.18]	26.5 ± 3.3<br>[33 ± 5.1]
AsSug408 (mg As/kg algae d.m.)	0.076 ± 0.06<br>[0.67 ± 0.53]	< 0.01
DMA (mg As/kg algae d.m.)	0.023 ± 0.009<br>[0.21 ± 0.10]	1.58 ± 0.14<br>[2.0 ± 0.35]
I-As (mg As/kg algae d.m.)	0.13 ± 0.17<br>[1.1 ± 1.6]	< 0.01

Table S5: total arsenic species determined in the lipophilic fraction, in *C. reinhardtii* (CR, n=12) cultivated at two different time points under identical conditions, *S. latissima* (SL) (n=3); [% of total As]

	CR	SL
AsPhytol (mg As/kg algae d.m.)	2.7 ± 0.5<br>[25 ± 5.4]	-
AsSugPL (mg As/kg algae d.m.)	1.4 ± 0.9<br>[12 ± 9.0]	1.2 ± 0.030<br>[1.4 ± 0.13]
mAsSugPL (mg As/kg algae d.m.)	0.39 ± 0.15<br>[3.6 ± 1.5]	0.48 ± 0.03<br>[0.60 ± 0.033]
AsFA (mg As/kg algae d.m.)	-	0.071 ± 0.005<br>[< 0.1]
AsHC (mg As/kg algae d.m.)	-	0.17 ± 0.007<br>[0.21 ± 0.015]
Hydrophilic As in this fraction (mg As/kg algae d.m.)	1.8 ± 1.1<br>[16 ± 10]	2.1 ± 0.29<br>[2.6 ± 0.12]

Table S6: Identification of As-lipids by ESMS and MSMS (mass accuracy and elemental composition) in *S. latissima* and *C. reinhardtii*

compound	elemental composition	FA chain length	theoretical [M+H]+	*S. latissima*	*C. reinhardtii*	RT (min)
				Δppm	Δppm	
**AsFA**						
AsFA422	$C_{22}H_{35}AsO_3$	C20:5	423.188	-1.5		18.4
AsFA424	$C_{22}H_{37}AsO_3$	C20:4	425.203	-0.5		18.6
**AsHC**						
AsHC360$^+$	$C_{19}H_{41}AsO$	C17:0	361.245	-0.3		21.2
AsHC374	$C_{20}H_{43}AsO$	C18:0	375.26	2.1*		21.5
AsHC388$^+$	$C_{21}H_{45}AsO$	C19:0	389.276	-1.1		21.7
**AsSugPhytol**						
AsSugPhytol544	$C_{28}H_{53}AsO_5$	C20:2	545.318		-0.1*	21.7
AsSugPhytol546$^+$	$C_{28}H_{55}AsO_5$	C20:1	547.334		-0.4	21.9
AsSugPhytol562-O	$C_{28}H_{55}AsO_6$	C20:1(OH)	563.329		0.5	21.7
**AsSugPL**						
AsSugPL930$^+$	$C_{43}H_{84}AsO_{14}P$	C16:0/C14:0	931.489	2.0		26.9
AsSugPL944	$C_{44}H_{86}AsO_{14}P$	C16:0/C15:0	945.504	3.0*		27.8
AsSugPL958$^+$	$C_{45}H_{88}AsO_{14}P$	C16:0/C16:0	959.52	1.7	1.6	28.8
AsSugPL972$^+$	$C_{46}H_{90}AsO_{14}P$	C16:0/C17:0	973.536	1.3*		30.2
AsSugPL986$^+$	$C_{47}H_{92}AsO_{14}P$	C16:0/C18:0	987.551	1.7	1.6*	31.6
AsSugPL1000	$C_{48}H_{94}AsO_{14}P$	C16:0/C19:0	1001.57	-1.7*	-0.3*	
AsSugPL1014$^+$	$C_{49}H_{96}AsO_{14}P$	C16:0/C20:0	1015.58	0.9		35.5
AsSugPL1028	$C_{50}H_{98}AsO_{14}P$	C16:0/C21:0	1029.6	-3.0*		38.0
AsSugPL984$^+$	$C_{47}H_{90}AsO_{14}P$	C16:0/C18:1	985.536	2.7*	1.1	29.1
AsSugPL982$^+$	$C_{47}H_{88}AsO_{14}P$	C16:0/C18:2	983.52	1.8*	1.3	27.5
AsSugPL980$^+$	$C_{47}H_{86}AsO_{14}P$	C16:0/C18:3	981.5044		-1.3*	26.5
AsSugPL956$^+$	$C_{45}H_{86}AsO_{14}P$	C16:0/C16:1	957.504	2.2*	0.6*	27.1
AsSugPL954$^+$	$C_{45}H_{84}AsO_{14}P$	C16:0/C16:2	955.489	2.5*	0.9*	25.9
**mAsSugPL**						
mAsSugPL692	$C_{27}H_{54}AsO_{13}P$	C14:0	693.259	0.5		21.3
mAsSugPL706	$C_{28}H_{56}AsO_{13}P$	C15:0	707.275	0.6*		21.7
mAsSugPL720$^+$	$C_{29}H_{58}AsO_{13}P$	C16:0	721.29	0.7*	0.5	21.9
mAsSugPL720$^+$	$C_{29}H_{58}AsO_{13}P$	C16:0	721.29	1.2*		22.1
mAsSugPL734$^+$	$C_{30}H_{60}AsO_{13}P$	C17:0	735.306	1.1*		22.4
mAsSugPL748$^+$	$C_{31}H_{62}AsO_{13}P$	C18:0	749.322	1.4		22.7
mAsSugPL776$^+$	$C_{33}H_{66}AsO_{13}P$	C21:0	777.353	0.7*		23.4

+: MeOH-adduct was present as well; *: MS-signal not intense enough for fragmentation, compound composition deduced from accurate mass MS, co-eluting arsenic (ICPMS/MS) and comparison with literature

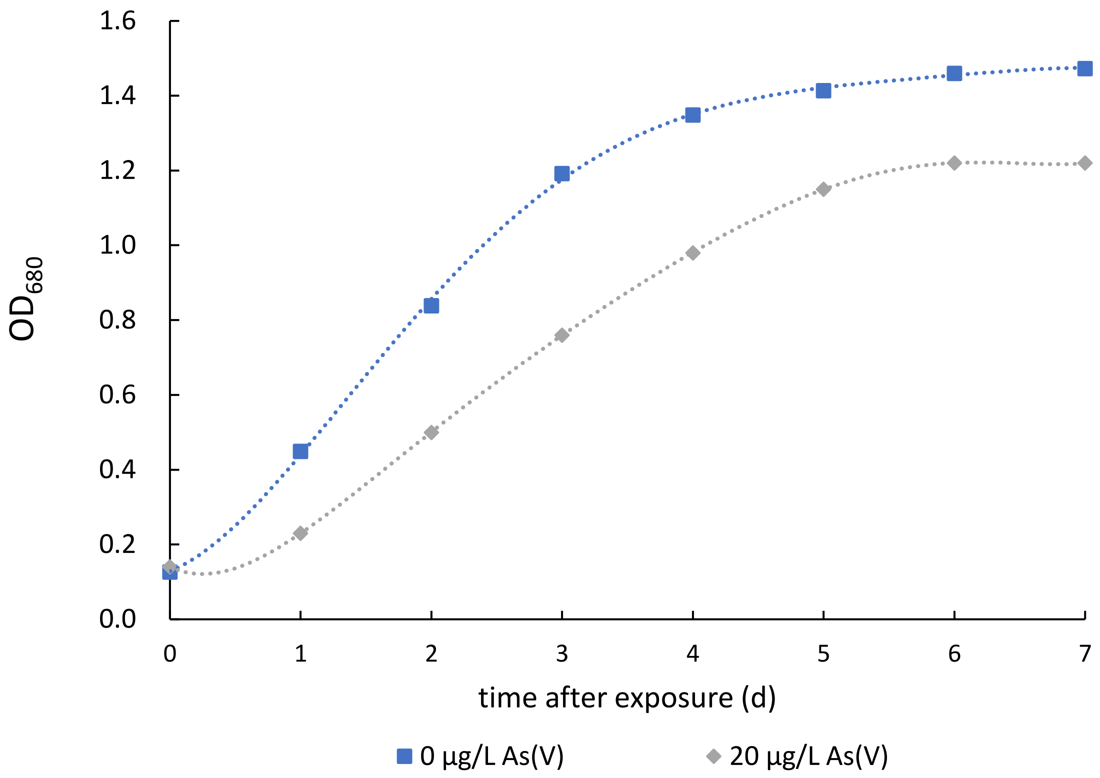

Figure S1: Growth curves over 7 days as determined by OD_{680} for *C. reinhardtii* with and without As(V) addition

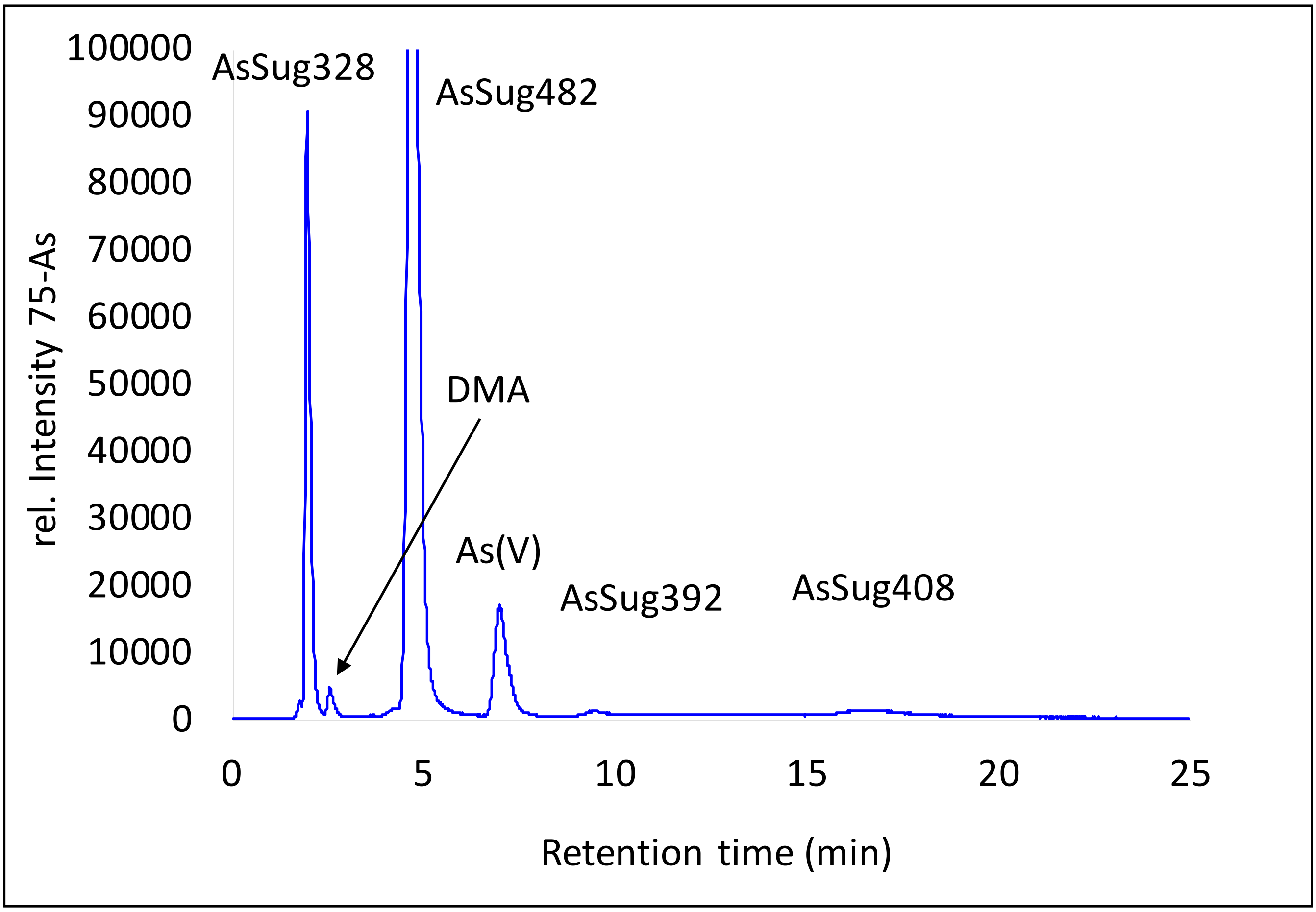

Figure S2: separation of hydrophilic arsenic compounds present in *C. reinhardtii* separated on an anion exchange column using 20 mM $NH_4H_2PO_4$ buffer pH 6.02 (average of 6 extracts/separations) using HPLC-ICPMS.

MS and MS/MS spectra of compounds identified in SL by qTOF-MS

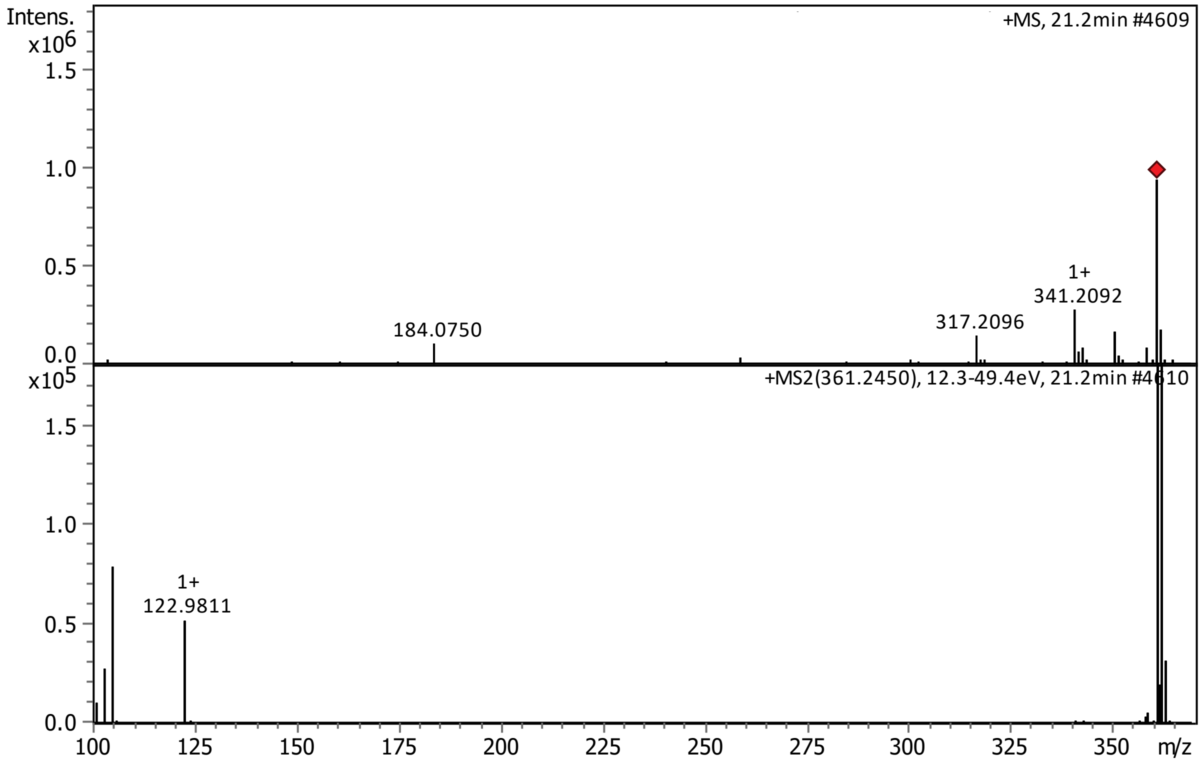

Figure S3: ESI-q-TOF MS and MSMS spectra of AsHC360 ([M+H]$^+$ 361.2450) in *S.latissima*

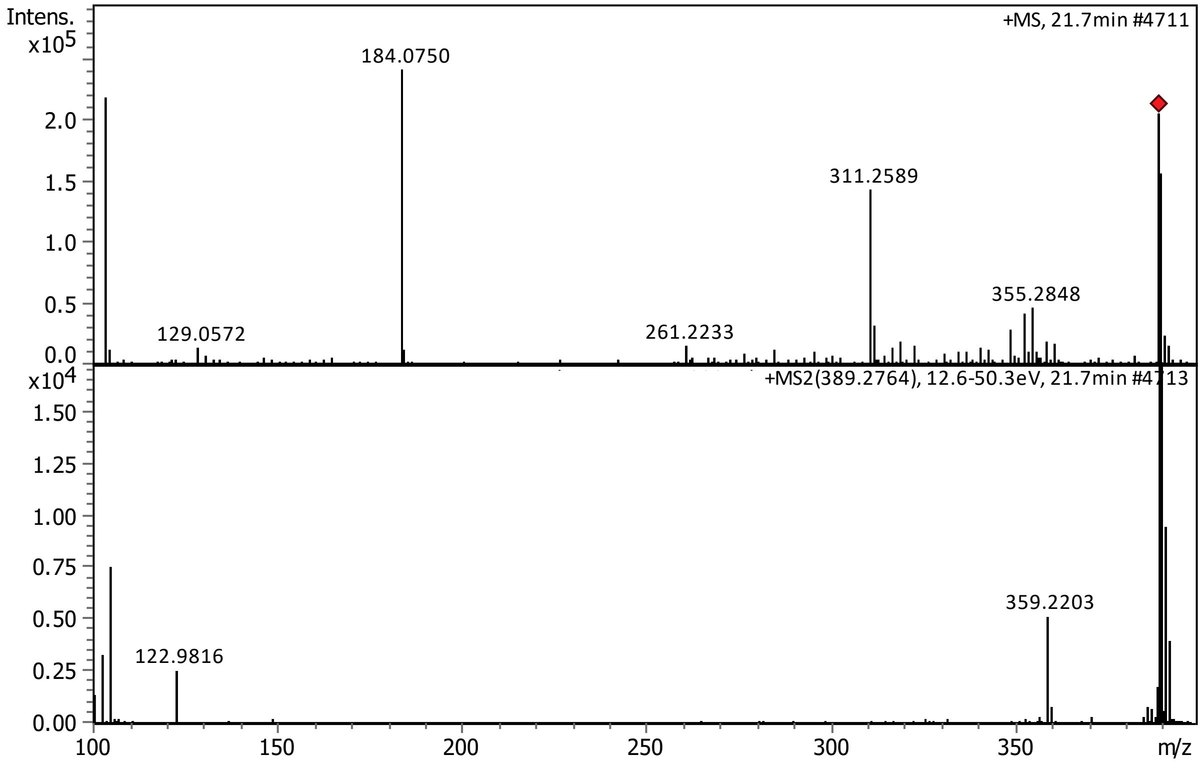

Figure S4: ESI-q-TOF MS and MSMS spectra of AsHC388 ([M+H]$^+$ 389.276) in *S.latissima*

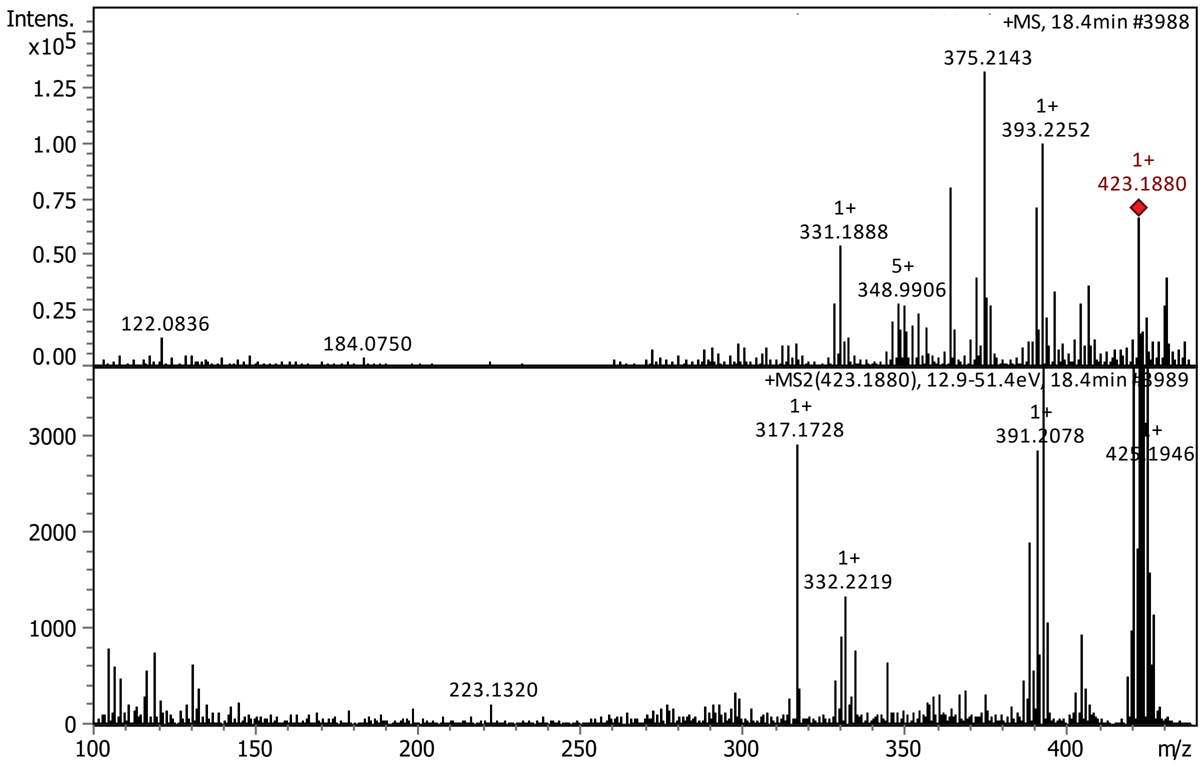

Figure S5: ESI-q-TOF MS and MSMS spectra of AsFA422 ($[M+H]^+$ 423.188) in *S.latissima*

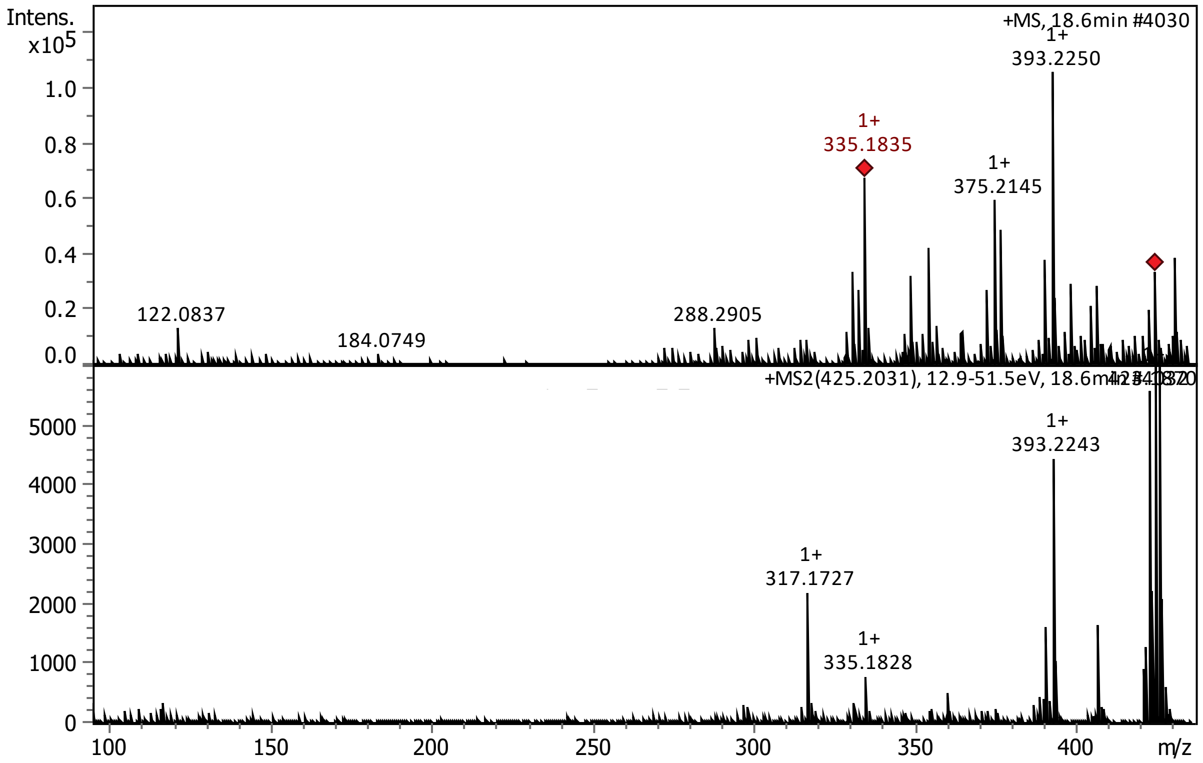

Figure S6: ESI-q-TOF MS and MSMS spectra of AsFA424 ($[M+H]^+$ 425.203) in *S.latissima*

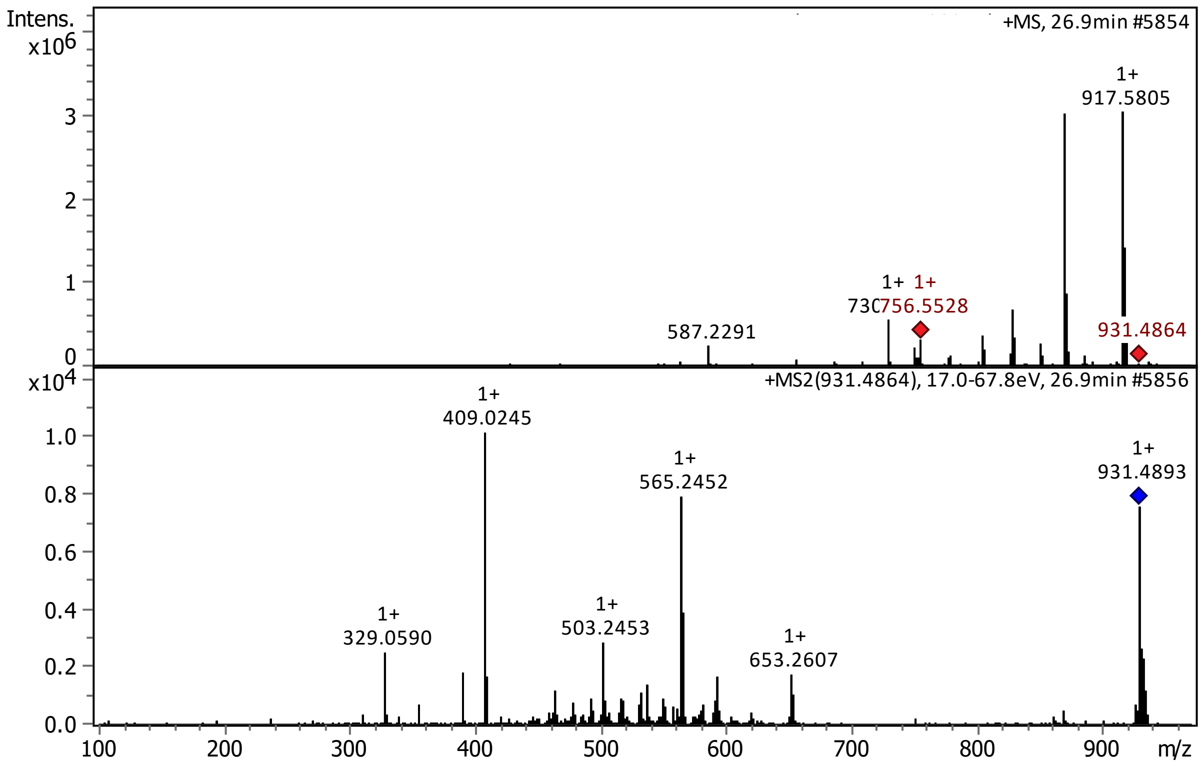

Figure S7: ESI-q-TOF MS and MSMS spectra of AsSugPL930 ([M+H]$^+$ 931.4864) in *S.latissima*

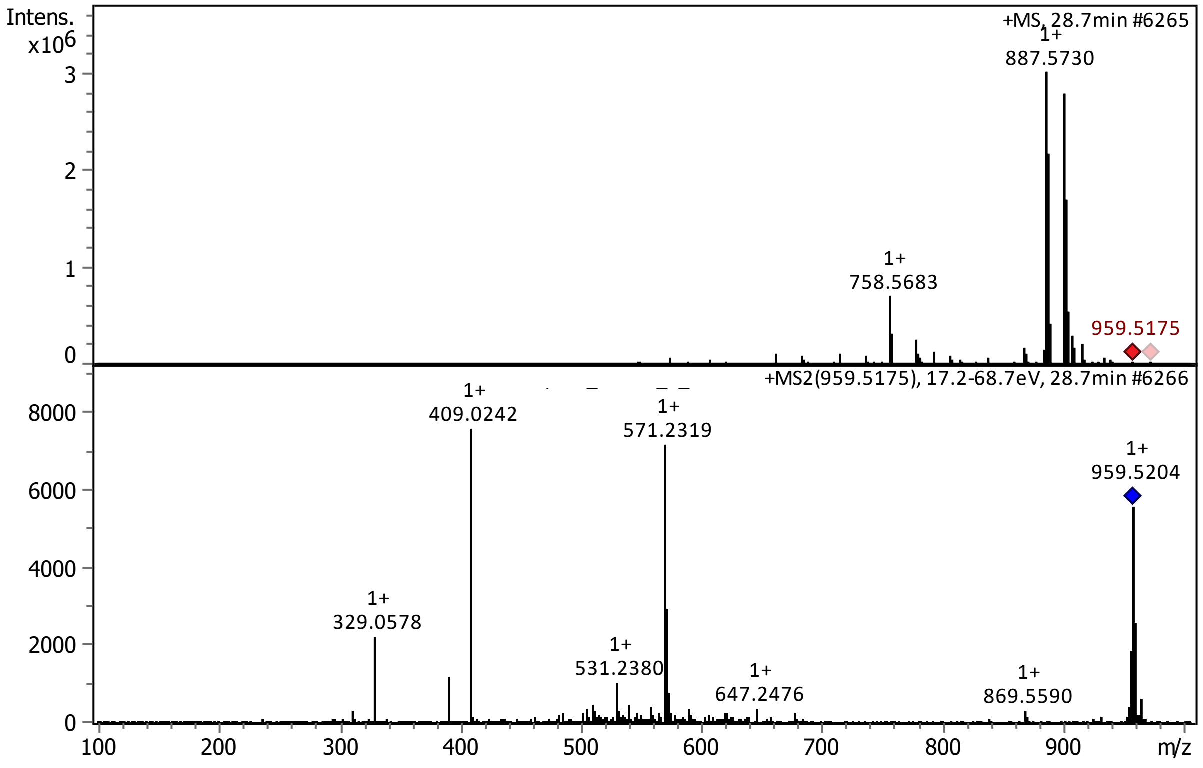

Figure S8: ESI-q-TOF MS and MSMS spectra of AsSugPL958 ([M+H]$^+$ 599.5175) in *S.latissima*

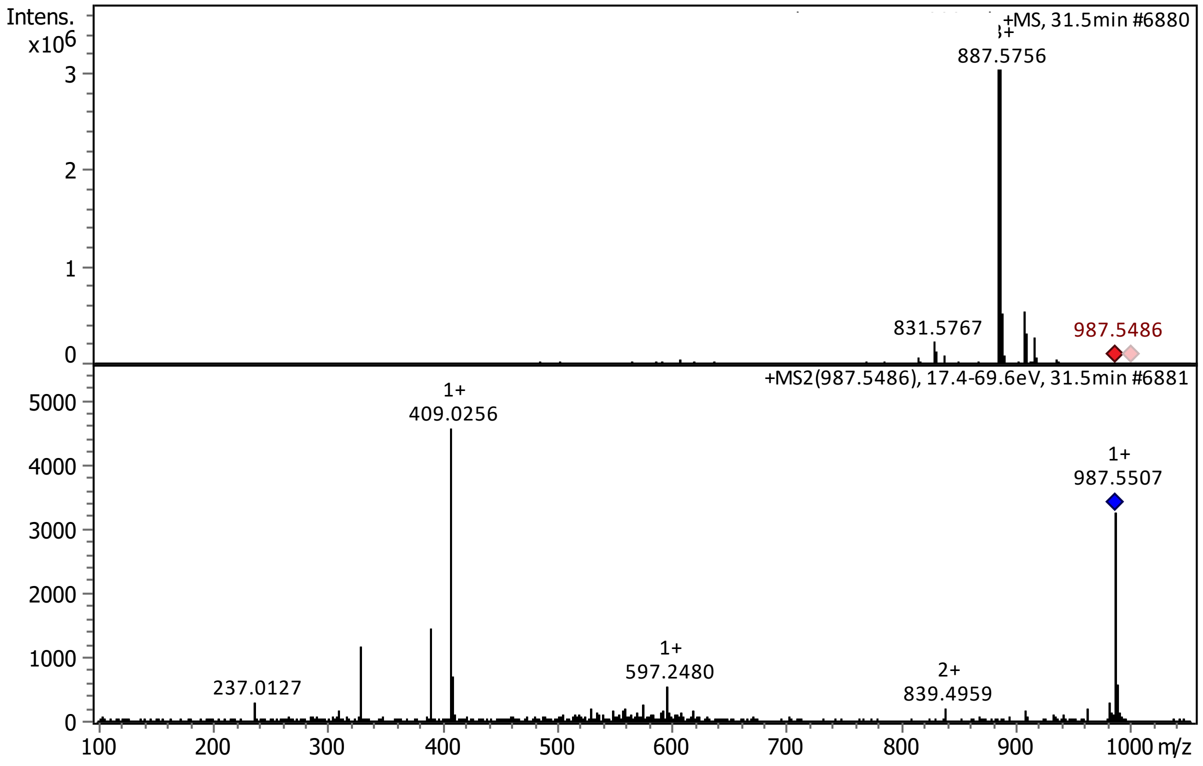

Figure S9: ESI-q-TOF MS and MSMS spectra of AsSugPL986 ([M+H]$^+$ 987.5486) in *S.latissima*

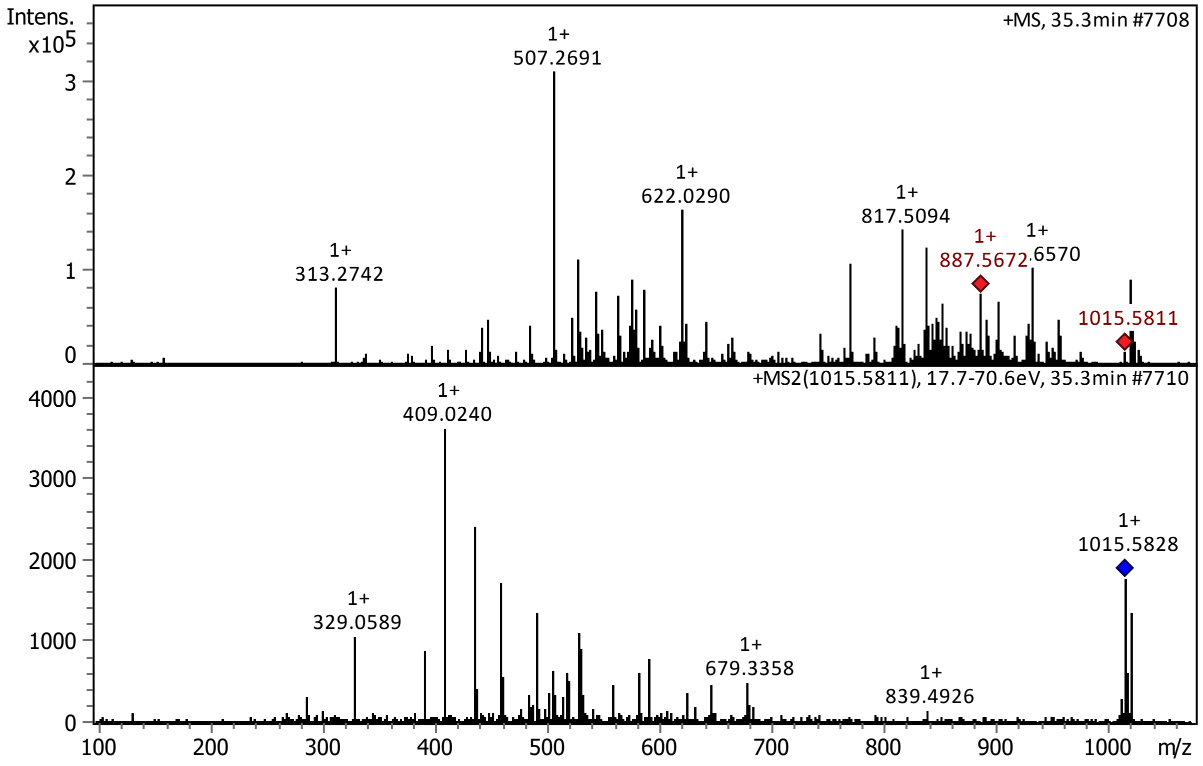

Figure S10: ESI-q-TOF MS and MSMS spectra of AsSugPL1014 ([M+H]$^+$ 1015.5811) in *S.latissima*

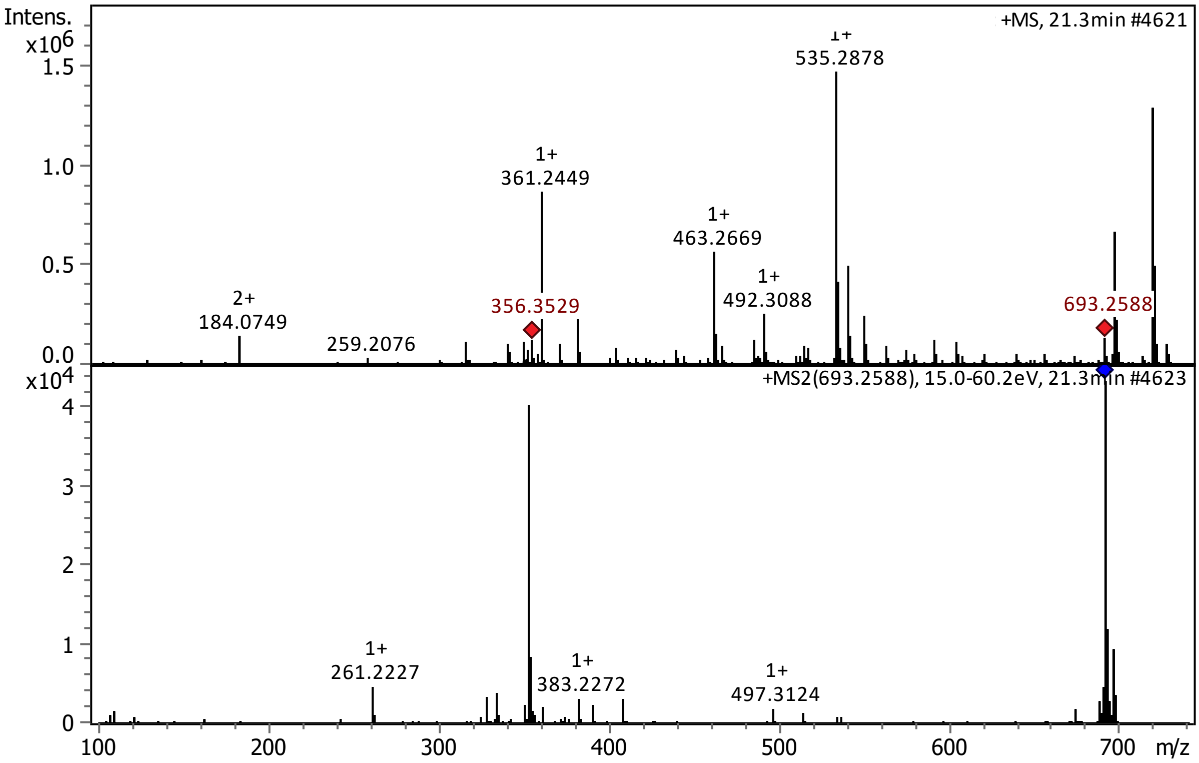

Figure S11: ESI-q-TOF MS and MSMS spectra of mAsSugPL692 ([M+H]$^+$ 693.2588) in *S.latissima*

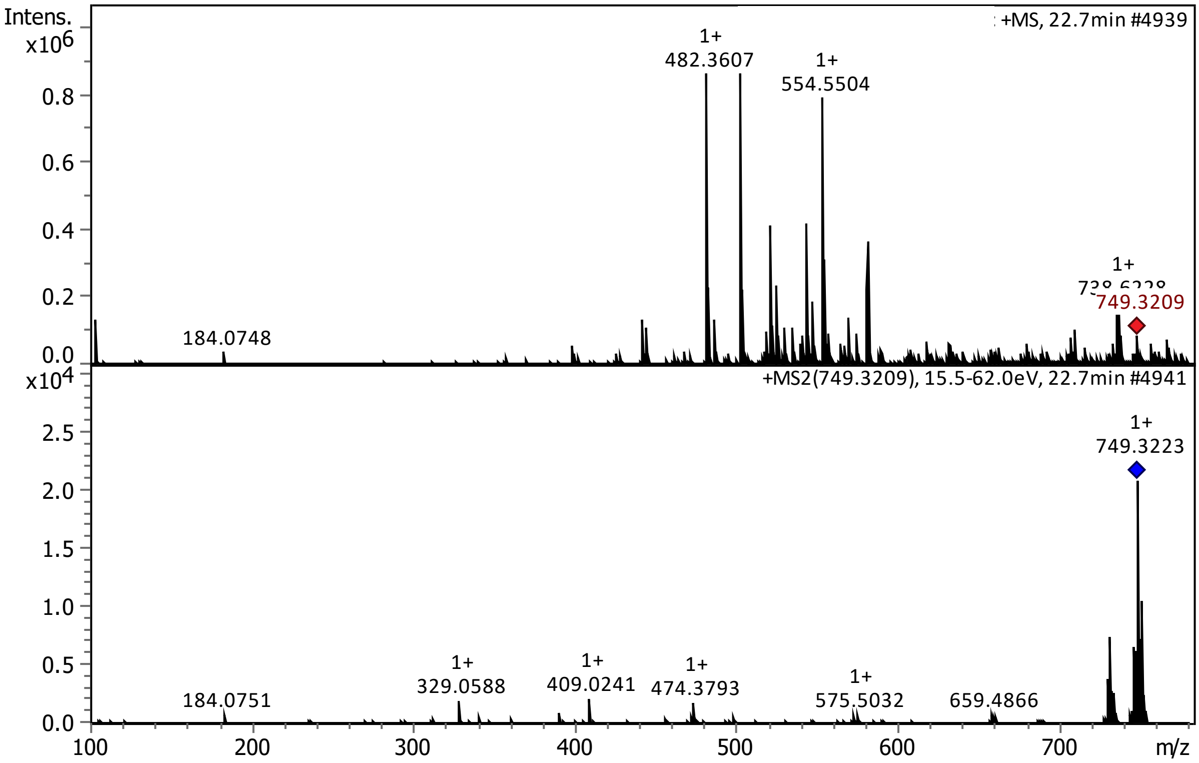

Figure S12: ESI-q-TOF MS and MSMS spectra of mAsSugPL748 ([M+H]$^+$ 749.3209) in *S.latissima*

MS and MS/MS spectra of compounds identified in CR by qTOF-MS

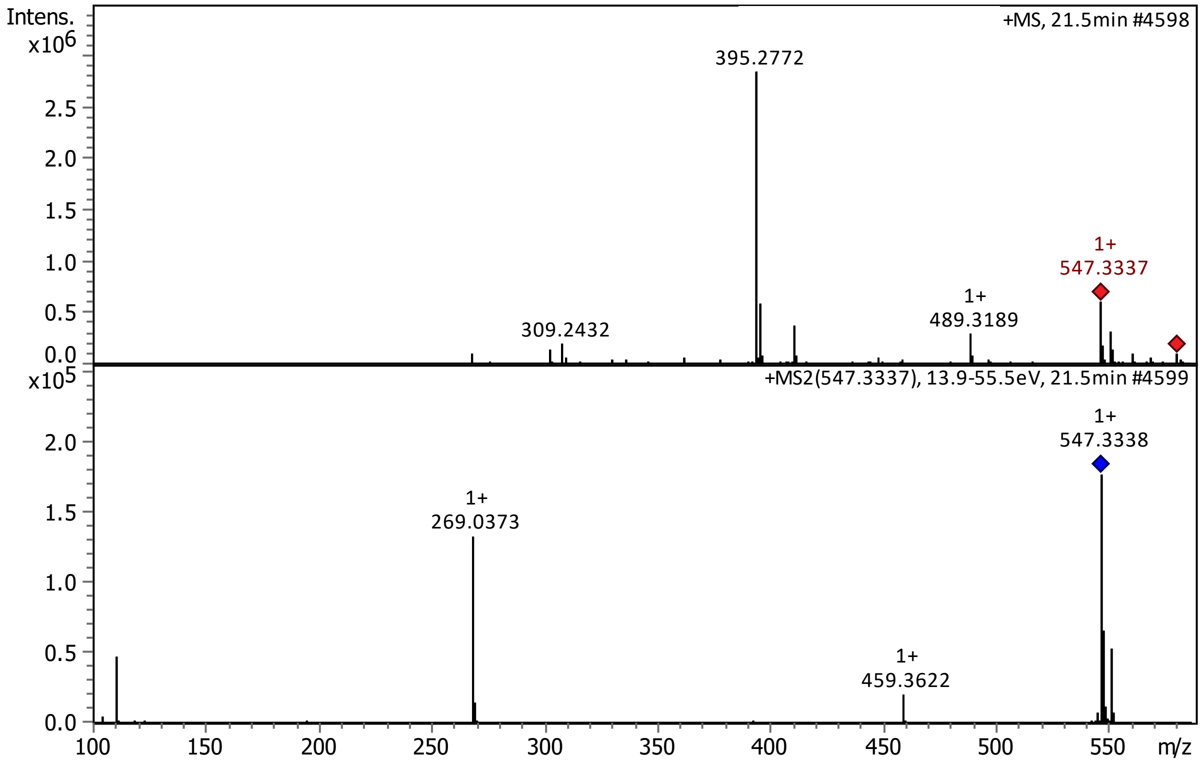

Figure S13: ESI-q-TOF MS and MSMS spectra of AsSugPhytol546 ($[M+H]^+$ 547.3337) in *C.reinhardtii*

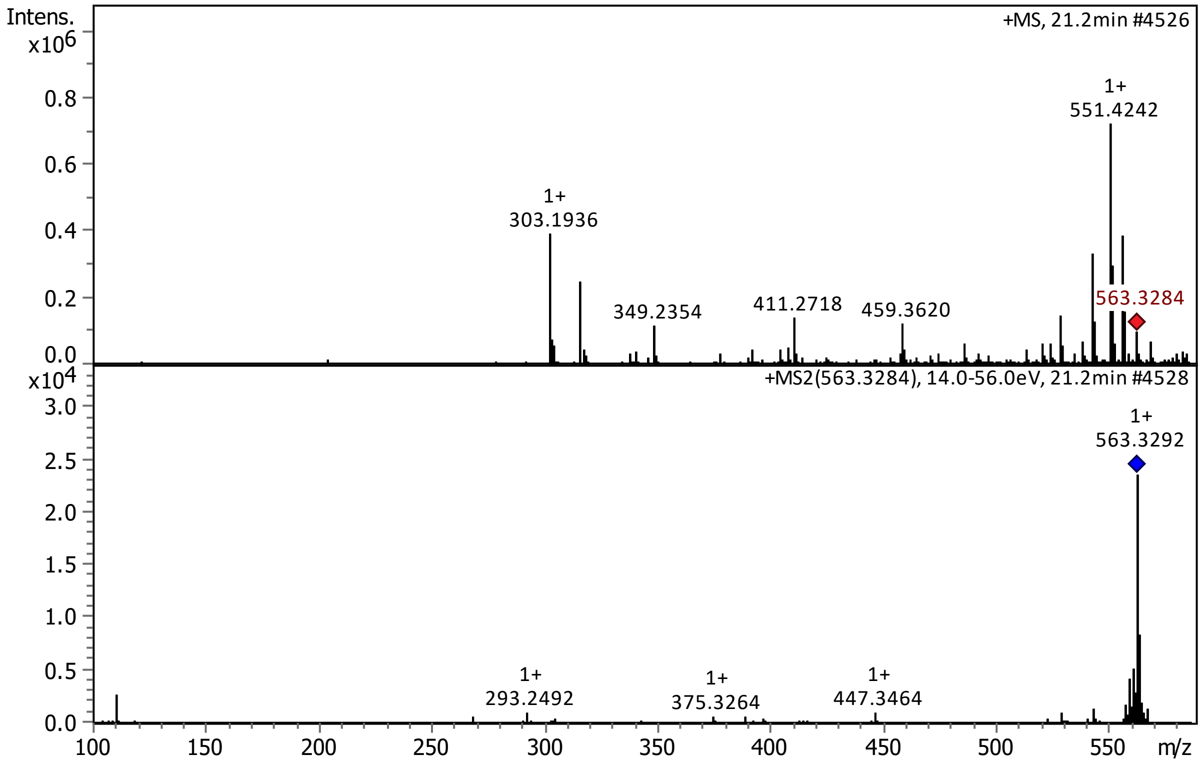

Figure S14: ESI-q-TOF MS and MSMS spectra of AsSugPhytol562 ($[M+H]^+$ 563.3284) in *C.reinhardtii*

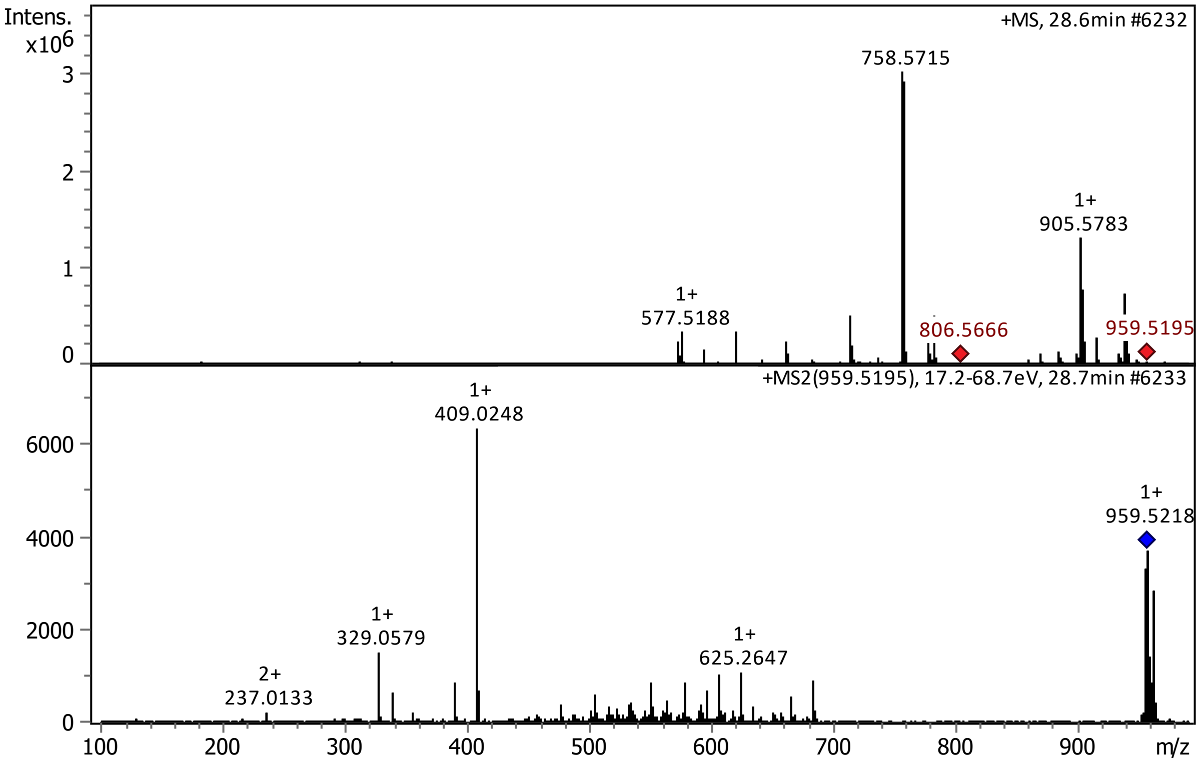

Figure S15: ESI-q-TOF MS and MSMS spectra of AsSugPL958 ([M+H]$^+$ 959.5195) in *C.reinhardtii*

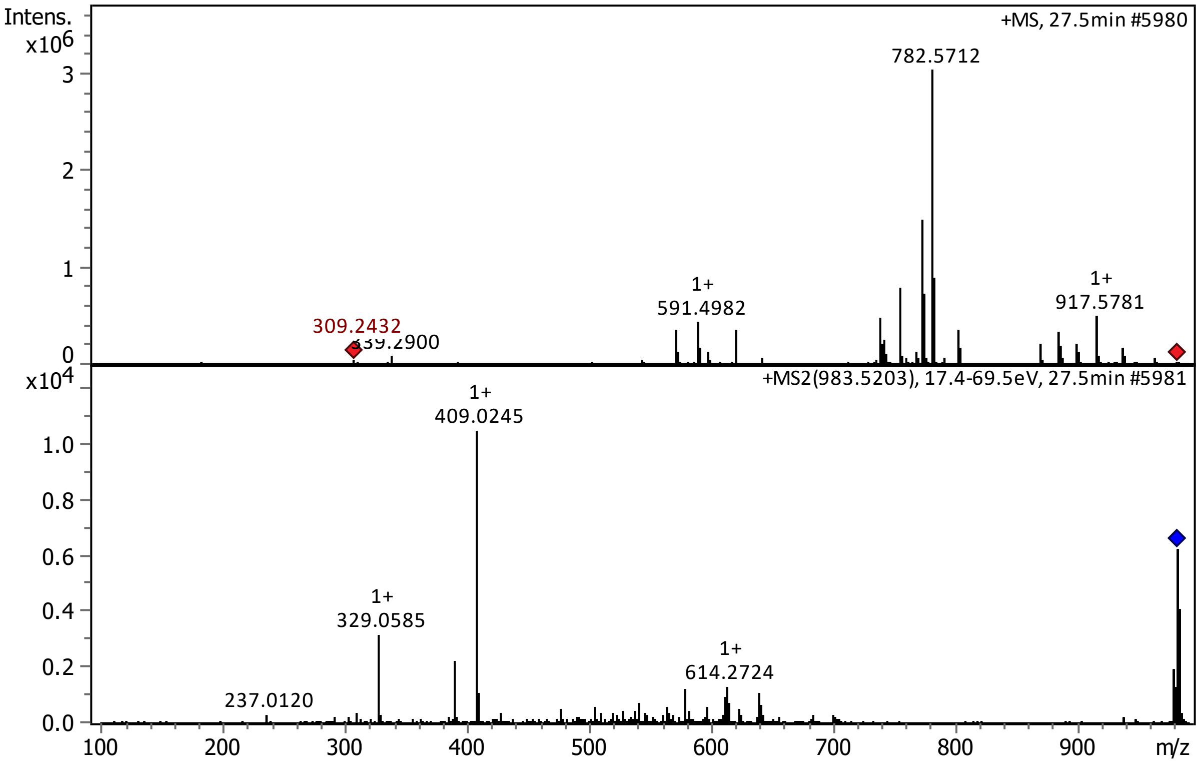

Figure S16: ESI-q-TOF MS and MSMS spectra of AsSugPL982 ([M+H]$^+$ 983.5203) in *C.reinhardtii*

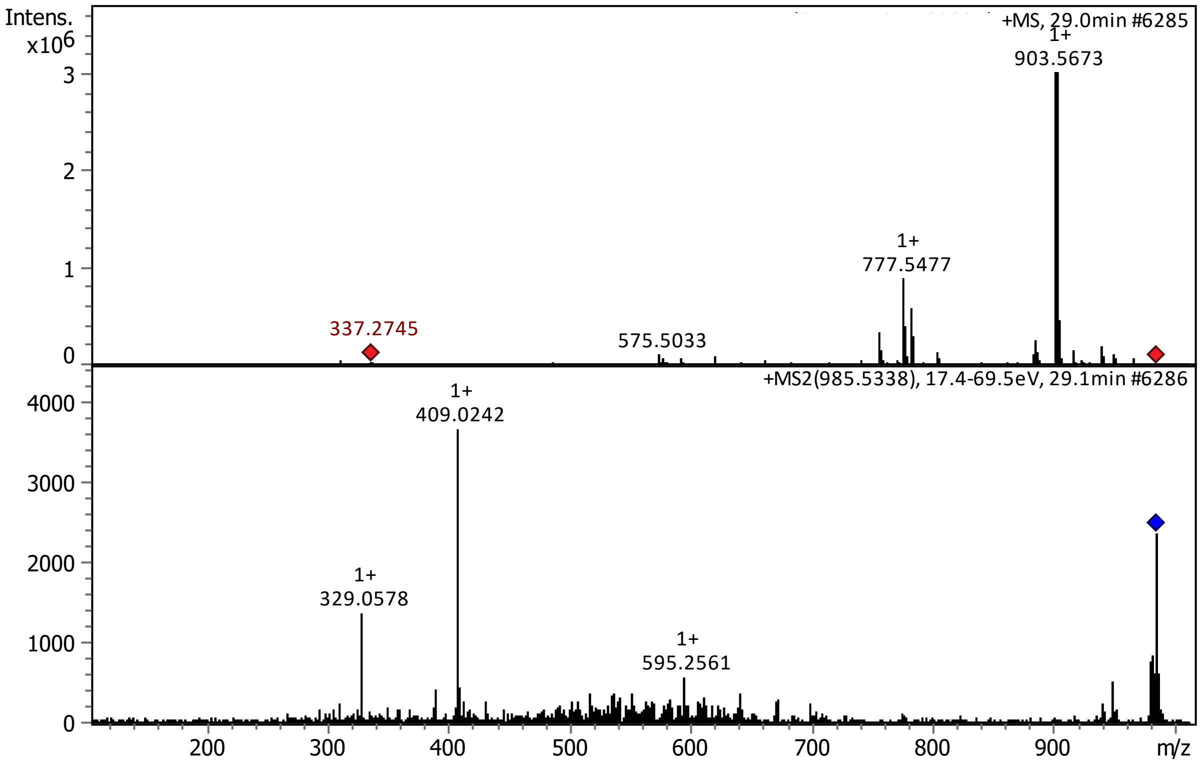

Figure S17: ESI-q-TOF MS and MSMS spectra of AsSugPL984 ([M+H]$^+$ 985.5338) in *C.reinhardtii*

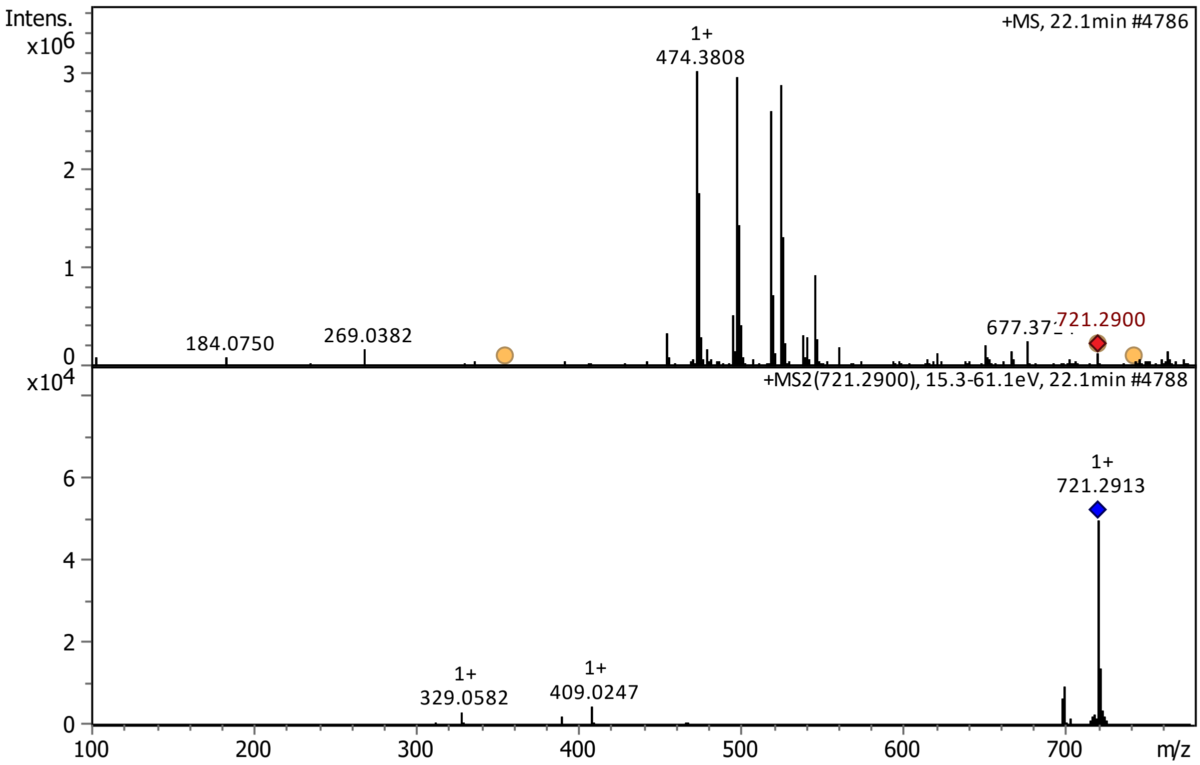

Figure S18: ESI-q-TOF MS and MSMS spectra of mAsSugPL720 ([M+H]$^+$ 721.2900) in *C.reinhardtii*
